# Immunologic treatments for precancerous lesions and uterine cervical cancer

**DOI:** 10.1186/1756-9966-33-29

**Published:** 2014-03-26

**Authors:** Patrizia Vici, Luciano Mariani, Laura Pizzuti, Domenico Sergi, Luigi Di Lauro, Enrico Vizza, Federica Tomao, Silverio Tomao, Claudia Cavallotti, Francesca Paolini, Aldo Venuti

**Affiliations:** 1 Department of Medical Oncology B, Regina Elena National Cancer Institute, V Elio Chianesi 53, Rome 00144, Italy; 2 Department of Gynecologic Oncology, Regina Elena National Cancer Institute, V Elio Chianesi 53, Rome 00144, Italy; 3 HPV Unit, Regina Elena National Cancer Institute, V Elio Chianesi 53, Rome 00144, Italy; 4 Department of Gynaecology and Obstetrics, “La Sapienza” University, V Policlinico 155, Rome 00161, Italy; 5 Department of Medical-Surgical Sciences and Biotechnologies, “La Sapienza” University of Rome, Oncology Unit, C.so della Repubblica, Latina 04100, Italy; 6 Department of Cinical Dermatology, San Gallicano Dermatologic Institute, V Elio Chianesi 53, Rome 00144, Italy; 7 Laboratory of Virology, HPV Unit, Regina Elena National Cancer Institute, V. Elio Chianesi 53, Rome, Italy

**Keywords:** Therapeutic vaccines, Cervical cancer, CIN, HPV, High-Grade SIL, Low-Grade SIL

## Abstract

Development of HPV-associated cancers not only depends on efficient negative regulation of cell cycle control that supports the accumulation of genetic damage, but also relies on immune evasion that enable the virus to go undetected for long periods of time. In this way, HPV-related tumors usually present MHC class I down-regulation, impaired antigen-processing ability, avoidance of T-cell mediated killing, increased immunosuppression due to Treg infiltration and secrete immunosuppressive cytokines. Thus, these are the main obstacles that immunotherapy has to face in the treatment of HPV-related pathologies where a number of different strategies have been developed to overcome them including new adjuvants. Although antigen-specific immunotherapy induced by therapeutic HPV vaccines was proved extremely efficacious in pre-clinical models, its progression through clinical trials suffered poor responses in the initial trials. Later attempts seem to have been more promising, particularly against the well-defined precursors of cervical, anal or vulvar cancer, where the local immunosuppressive milieu is less active. This review focuses on the advances made in these fields, highlighting several new technologies (such as mRNA vaccine, plant-derived vaccine). The most promising immunotherapies used in clinical trials are also summarized, along with integrated strategies, particularly promising in controlling tumor metastasis and in eliminating cancer cells altogether.

After the early promising clinical results, the development of therapeutic HPV vaccines need to be implemented and applied to the users in order to eradicate HPV-associated malignancies, eradicating existing perception (after the effectiveness of commercial preventive vaccines) that we have already solved the problem.

## Introduction

Cervical cancer is the third most common cancer in women and the fifth most common overall cancer worldwide as age standardized incidence rate in both sexes combined [1]. The prime causal factor of the disease is a persistent infection with high-risk human papillomavirus (HPV), with individuals failing to mount a sufficient immune response against the virus. The high-risk HPV genome encodes three oncoproteins, E5, E6 and E7, the last two oncoproteins are constitutively expressed in high-grade lesions and cancer, and are required for the onset and maintenance of the malignant phenotype. About 170 HPV genotypes have been identified and 40 can infect the ano-genital area: uterine cervix, vulva, vaginal wall, penis and anus. HPVs are classified as high-risk types, commonly associated with cancer, and low-risk, mostly identified in condyloma acuminatum. The International Agency for Research on Cancer (IARC) conducted a study on over 30,000 cervical cancers that showed HPV 16, 18, 58, 33, 45, 31, 52, 35, 59, 39, 51, 56, to be the most common types associated with invasive cervical cancer with HPV 16 accounting for over 50% and HPV 16 and 18 for >70% worldwide [2]. Epidemiological data report that HPV infection occurs at least once during lifespan in about 75% of U.S. women [3], and natural history shows that most HPV infections resolve spontaneously, while in some women infection persists and progresses to cervical cancer. The incidence of high-grade cervical intraepithelial neoplasia (CIN 3) is about 1-2 per 10 females with low-grade CIN, and without treatment about one third progresses to cervical cancer [4,5].

Cervical carcinogenesis is a multi-step process, which starts with viral infection and requires the establishment of persistent HPV infection. Papillomavirus persistence is favored by at least three major factors: the virus life cycle that takes place away from dermal immune cells, the absence of virus-induced cell lysis with, in turn, no or weak inflammatory response and finally the altered immune response induced by viral proteins. Thus, cancer development depends not only on efficient negative regulation of cell cycle control supporting the accumulation of genetic damage, but also on immune evasion that enables the virus to lie undetected for a long time. Many studies confirm that persistent HPV infection is the most important risk factor, mainly with high-risk subtypes [5]. Studies in HIV women or in patients treated with immunosuppressive agents reported an increased incidence of CIN lesions, suggesting an important role of cell-mediated immune response against HPV antigens [6,7]. The role of systemic and local mucosal immune responses to HPV antigens is controversial. Some studies suggest a positive association between systemic cell-mediated immune responses and the regression of CIN [8]. Moreover, antibody responses to the major viral capsid protein, L1, can be detected by about 6 months after infection and may be observed up to 5 years later in women who have been cleared from infection. Type specific L1 antibody responses have also been detected in persistent disease and cancer in about half of the patients [9,10].

A number of escape factors may affect the natural immune response against HPV proteins, together with the loss of correct signals from immune system to activate adaptive immune system. Indeed, optimal activation of adaptive immunity and generation of specific CD4 T helper 1 type immunity supporting development of CD8 cytotoxic T cells against viral early proteins, like E2, E6, E7, is critical for virus clearance in basal epithelial cells. T helper cells also support optimal activation of B cells, with secreting HPV capsid type specific neutralizing antibodies, which can protect against subsequent infections at mucosal and systemic levels [11]. Spontaneous regression mainly occurs in lesions infiltrated by CD4+ and cytotoxic CD8+ T cells and it is also associated with circulating HPV early antigen-specific CD4+ and CD8+ T cells, which also have a favorable prognostic significance [12-15].

In the early phases of carcinogenesis the three oncogenes of the virus E5, E6 and E7 play an important role in immune evasion. In particular, the E5 protein [16] seems to further facilitate the virus-induced immune escape by down-regulating MHC/HLA class I and II [17,18] and inducing a reduction in recognizing CD8+ T cells, at least *in vitro*[19]. This down regulation does not affect the HLA molecules (HLA-C/E) whose presence on the cell surface to avoid natural killer action is essential [20,21]. Several pieces of experimental evidence show that neither synthesis nor transport to the cell surface of HLA-C/E is affected by E5 expression, leading to the conclusion that E5 selectively inhibits surface expression of HLA-A and HLA-B [17]. In this manner, high risk HPVs are potentially capable of avoiding both CTL and NK cell killing, favoring persistent infection. In the late phases, when integration of the virus (frequently with loss of E2 and E5 genes) into the host genome blocks the productive life cell cycle, favoring immortalization and mutation acquisition, the E6 and E7 still play a fundamental role: i) high-risk E6 reduces the surface expression of CDH1 by epithelial cells; ii) E6 and E7 inhibit the transcription of toll-like receptor (TLR) 9, necessary to activate antigen-presenting cells as part of innate immune response; iii) E7 reduces expression of transporter associated with antigen processing 1 (TAP1), a component of the presentation and processing pathway, so blocking the activation of specific T lymphocytes; iv) high-risk HPVs down-regulates the expression of pro-inflammatory cytokines [22].

In addition, therapeutic T cell effector mechanisms are limited due to the following: changes in local immunity; the production of cytokines such as Interleukin (IL)-10 or transforming growth factor (TGF-beta); increased number of regulatory T cells (Tregs) and to immunosuppressive myeloid cells that are commonly observed in HPV related cancers. Moreover, frequent mutational events in cancer include HLA loss of expression, with subsequent escape of tumor cells [23,24].

In this manner, HPV-related tumors usually present MHC class I down-regulation, impaired antigen-processing ability, avoidance of T-cell mediated killing, increased immunosuppression due to Treg infiltration, and secrete immunosuppressive cytokines [25].

These are the main obstacles faced when achieving a valid immunotherapy against HPV-related pathologies where a number of different strategies have been developed in order to overcome them including adjuvants. Adjuvant is a term derived from the Latin word, *adjuvare*, which means to aid or to help. Historical boosting humoral immune response was the goal of the developed adjuvants and, as a result, many commonly used adjuvants are effective in elevating serum antibody titers, but do not elicit significant Th1 responses or cytotoxic T lymphocytes (CTLs). However, certain adjuvants have recently been demonstrated to be able to induce cellular immunity which are summarized in Table 1 according to their mechanism of action [26].

**Table 1:** List of adjuvants by their dominant mechanism of action

**Antigen delivery systems**	**Immunopotentiators**
• Electroporation	• Alternative pathogen-associated molecular patterns (PAMPs): e.g. *Cholera* enterotoxin; liquenase.
• Gene gun	• Heat-shock proteins
• Liposomes	• Lysosome and endocelllular reticulum (ER) targeting agents
• Virosomes™	• Saponins (Quils, QS-21)
• ISCOMS®	• TLRs agonists: e.g, imiquimod, oligonucleotides (CpG, etc.), double-stranded RNA (dsRNA)
• Micro/Nanoparticles: e.g. microparticles of poly (lactide-co-glycolide) (PLG)	• Cytokines & chemokines: e.g. IL2, IL12 and GM-CSF
• Emulsions: e.g., MF59, Montanides	• Treg inactivators: e.g. anti-apoptotic molecules, low-dose cyclophosphamide, antibodies anti-CD 25, -CTLA, -IL10, or -PDL-1
• Virus-like particles & viral/bacterial vectors	• Monophosphoryl lipid A (MPL) and synthetic derivates
	• Muramyl dipeptide (MDP) and derivatives

Conceptually, immunity can be utilized in a therapeutic setting in two ways: first, by using specific natural o synthetic antibodies against defined targets, or second, by inducing an immune response in the organism against specific antigens (preventive and therapeutic vaccines). In particular, for HPV-induced lesions and cancer viral antigens or/and virus-induced host antigens can be targeted by these approaches. Indeed, once a patient is infected with HPV there is no effective way to cure persistent HPV infection which is the first step towards the development of pre-cancerous lesions. It was estimated that even with mass vaccination through commercially highly effective preventive quadrivalent or bivalent HPV vaccines [27-31], it will take at least 20 years before the incidence and prevalence of cervical cancer significantly decreases due to the slow rate of carcinogenesis. Furthermore, during this period of time many people will be infected by high risk HPV that will consequently increase the burden of established HPV infections and HPV-associated diseases that lie undetected, untreated and slowly progressing toward malignant transformation.

Thus, there is great need for the development of novel therapeutic intervention to control HPV-associated disease and cancers. Given that existing treatments [32-34] are partially effective in cancers or pre-neoplastic lesions, and useless in persistent infections, immune therapies may represent a valid tool.

The following paragraphs summarize these issues and focus on the treatment (clinical trials) of already established infections (Table 2) or cancer (Table 3) where preventive intervention by vaccine has not effect.

**Table 2:** Clinical trials for HPV-associated pre-neoplastic lesions

**Vaccine**	**Antigen(s)**	**Phase**	**Lesions**
ADXS11-001:	HPV-16 E7	II	CIN 2/3, VIN 3, VAIN 2
Lm secreting fusion/LLO-HPV-16 E7 protein (Lm-LLO-E7)			
Procervix: adenylate cyclase protein vector delivering HPV16 and HPV18 E7 antigens	HPV-16 and HPV-18 E7	I/II	High-risk HPV infections before CIN appearance
MVA E2: Recombinant Modified Vaccinia Ankara (MVA) encoding E2 from BPV	Bovine Papillomavirus E2	I/II	CIN1-3
		I/II	Male flat condyloma
		II	High-grade CIN
TG4001/R3484:	HPV-16 E6/E7	IIa	CIN2/3
Recombinant MVA expressing E6-E7of HPV-16, and IL-2		IIb	
Peptides: HPV E7 (aa 12–20) plus E7 lipopetide (PADRE helper peptide, linker peptide, and E7 peptide: aa 86–93) & Montanide ISA-51 adjuvant	HPV-16 E7	I	High-grade CIN and
		II	VIN
		II	VIN 3
			HSIL
HPV-16 E6/ E7 fusion protein plus ISCOMATRIX adjuvant	HPV-16 E6 and E7	I	CIN 1–3, HPV-associated AIN in HIV-positive male
PD-E7: Modified HPV-16 E7/Hib protein D fusion protein & AS02B adjuvant	HPV-16 E7	I/II	CIN 1, CIN 3
SGN-00101: HPV-16 E7/M. bovis, Hsp65 fusion protein	HPV-16 E7	I/II	Anal HSIL
		I/II	High-grade AIN
		II	ASCUS and LSIL, recurrent respiratory papillomatosis, high-grade CIN
SGN-00101 in poly ICLC adjuvant	HPV-16 E7	I	CIN 1-3
ZYC101: Recombinant HPV-16 E7 DNA Plasmid encapsulated in poly microparticles	HPV-16 E7	I	High-grade AIN in males, CIN 2/3
ZYC101a: Recombinant HPV-16 and HPV 18 E6-E7 DNA Plasmid encapsulated in poly microparticles	HPV-16 and HPV-18 E6 and E7	II/III	High grade CIN
pNGVL4a-Sig/E7/Hsp70: DNA plasmid expressing mutated HPV-16 E7 fused to Sig and Hsp70	HPV-16 E7	I	CIN 2/3
pNGVL4a-CRT/E7: DNA plasmid expressing mutated HPV-16 E7 fused to calreticulin	HPV-16 E7	I	CIN 2/3
VGX-3100: DNA plasmid expressing HPV-16 and HPV-18 E6 and E7 proteins	HPV-16 and HPV-18 E6 and E7	I	CIN 2/3 (after surgery or fourth dose)
		II	CIN 2/3
TA-CIN/TA-HPV prime/boost	HPV-16 and HPV-18 E6 and E7 and HPV-16 L2	II	CIN 2/3
TA-HPV/TA-CIN prime/boost	HPV-16 and HPV-18 E6 and E7 and HPV-16 L2	II	CIN 2/3
pNGVL4a-Sig/E7 /Hsp70 and TA-HPV prime/boost plus TLR agonist imiquimod	HPV-16 and HPV-18 E6 and E7	II	CIN 2/3

Note: Abbreviations are as in the text.

**Table 3:** Clinical trials for HPV-associated cancer

**Vaccine**	**Antigen(s)**	**Phase**	**Cancer**
**ADXS11**-**001:**	HPV-16	I	Cervical cancer
**Lm secreting fusion to LLO-HPV-16 E7 protein (Lm-LLO-E7)**	E7	I	HPV- oropharyngeal cancer
		II	Recurrent cervical carcinoma
**TA**-**HPV: Recombinant vaccinia virus expressing E6 and E7 from both HPV-16 and HPV-18**	E6 and E7 of HPV16 and HPV-18 E6	I	Stage Ib and IIa cervical cancer
		I/II	Advanced cervical cancer
		II	Stage Ib and IIa cervical cancer
**PADRE peptide linked to E7 Lipopeptide**	HPV-16 E7	I	Cervical cancer
**HPV-16 E7 epitopes emulsified in Montanide ISA-51 adjuvant**	HPV-16 E7	I/II	Cervical cancer
**13 overlapping long peptides covering whole E6 and E7 sequences of HPV 16 plus Montanide ISA-51 adjuvant**	HPV-16 E6 and E7	I	Advanced cervical cancer stage Ib1 cervical cancer
		II	
**DC pulsed with HPV-16 E7**	HPV-16 E7	I	Recurrent cervical cancer
**pNGVL4a-Sig/E7/Hsp70: DNA plasmid expressing mutated HPV-16 E7 fused to Sig and Hsp70**	HPV-16 E7	I	Advanced HNSCC

Note: Abbreviations are as in the text.

## Therapeutic antibodies

The use of intracellular antibodies (intrabodies) to inhibit protein function holds promise for the treatment of human diseases. The difficulties associated with the development of intrabody-based therapies are similar to those that hamper the development of protein inhibitors in general, and reflect problems in generating reagents that are effective and specific. This effectiveness and specificity can be achieved by using intrabodies to combat intracellular parasites like viruses. Indeed, viruses express proteins that are directly involved in causing disease and are clearly diverse from those of the host cell. For viruses that cause only local infections, such as HPV, the intrabody approach may be more appropriate. Furthermore, not only the infected cells but also the transformed cells require the continuous expression of some HPV proteins, particularly the oncogenes E6 and E7. This is most evident in Hela cells, derived from an HPV-associated malignancy in the 1950s, which still almost 50 years later require the expression of these oncogenes for their growth in tissue culture [35,36].

Intrabodies against the E6 [37] and E7 [38] of HPV have been produced and proved effective in *in vitro* cancer cell models. More recently, an intrabody against the E7 of HPV 16 was proved to block tumor growth in animal models [39]. Given the accessibility of HPV-associated lesions to topical therapy, preclinical results suggest that large interfering molecules, such as intrabodies, may be useful inhibitors of viral protein–protein interactions and particularly appropriate for the treatment of HPV-associated diseases. However, no clinical study has been published so far.

Instead of using intrabodies, the utilization of monoclonal antibodies against membrane-expressed antigens is suggested, that may be induced by the HPV, i.e. Epidermal Growth Factor Receptor (EGFR). Monoclonal antibodies anti EGFR are already in clinical use and have been fully reviewed elsewhere [32]. However, beside HPV induction other membrane-associated antigens can be found in transformed cervical cells and may be targeted by monoclonal antibodies. An example of these antibodies is the adecatumum (MT201), a humanized monoclonal antibody targeting epithelial cell adhesion molecules, showing activity in cervical cancer cell lines over-expressing epithelial cell adhesion molecule (EpCAM) [40]. This activity may suggest a hypothetical clinical employment of this monoclonal antibody which has already been performed with monoclonal antibodies against EGFR.

## Therapeutic vaccines

Therapeutic vaccines aim to eradicate or reduce already infected cells by stimulating cytotoxic T cells against target infected cells and up-regulating MHC Class I expression. Vaccine-mediated immune strategies could be directed toward at least two different stages of the oncogenic infection: firstly, infection and then secondly, the established infection. By eliciting neutralizing antibody responses the prophylactic vaccines challenge the first infection by inhibiting the HPV to bind to the cell or the early phases of viral entry. These vaccines are already in clinical use and will not be discussed in this review.

The therapeutic vaccines should fight already infected cells, and could be tailored based on the presence of episomal replicating virus or integrated viral sequences. In the first case, the vaccine targets could be all the early proteins, in the second case only the E6-E7 proteins appear to be a realistic target of intervention [41]. In addition, as HPV 16 accounts for over 50% of invasive cancer worldwide, the clinical studies mainly focused on the E6 and E7 proteins of this HPV [2].

In the experimental model an effective immunotherapy administered before tumor challenge includes an antigen-specific component, whereas an effective immunotherapy after tumor challenge can be achieved through the enhancement of either innate or adaptive immunity, and seems to be optimal with both. Therefore, valid therapeutic vaccines must achieve this goal. Trials of immunotherapy in patients with HPV associated pre-malignancy are expected to be more effective than in cancer patients, since the impaired antigen presentation by cervical cancer cells due to mutations in MHC and TAP genes may render the immunotherapy less effective. Although there are potential immune-evasive mechanisms that are attributable to the HPV infection itself [42].

Examples of those therapeutic vaccines, which have reached phases I and II clinical trials, are presented in the subsequent paragraphs according to the different formulations.

### Protein/peptide-based vaccines

To date, several protein or peptide-based vaccines are either undergoing clinical evaluation or are in development. A major limitation to peptide-based vaccines is the HLA restriction that can be overcome by the use of whole protein-based vaccines, which harbor multiple immunogenic epitopes, binding various allelic HLA molecules. On the other hand, protein-based vaccines are generating predominantly antibody responses rather than CTL responses because proteins are processed through the endocytic/MHC class II pathway. In addition, both peptides and proteins are poorly immunogenic. Therefore, most of the research in this area was focused on the co-administration of adjuvant immune-enhancing agents such as chemokines, cytokines, and co-stimulatory molecules to enhance the potency of the vaccine. In particular saponin-based [43] or liposome–based (LPD) formulations [44], or TLR agonists [45] were employed as adjuvants for protein vaccines. Recently, the fusion of the beta-1,3-1,4-glucanase (LicKM) of Clostridium thermocellum bacterial protein to the HPV E7 protein produced an antigen with strong intrinsic adjuvating activity, indicating that this manipulation of the antigen may elicit some unknown helpful functions [46,47]. Many other fusion proteins were reported to elicit some adjuvating activities such as Mycobacteria-derived heat-shock proteins (Hsp) [48,49], truncated Pseudomonas aeruginosa exotoxin A [50], Bordetella pertussis adenylate cyclase [51], and the cell penetrating peptide Limulus polyphemus protein [52]. In addition, alternative delivery system such as electroporation can further improve the activity of this adjuvating protein vaccine [45].

Peptide-based vaccines need to increase not only the poor immunogenicity level but also the obstacle of MHC restriction. TLR agonists have also been explored as adjuvants for peptide-based HPV vaccines because of their capability to activate both innate and adaptive immunity. Vaccines consisting in CTL and or TH epitope adjuvated with TLR 9 [53], TLR4 or [54] and TLR3 [55] agonists demonstrated their efficacy in mouse models. This activity was demonstrated also by utilizing a CTL epitope fused to a T-helper epitope, pan-DR epitope (PADRE) [56]. These results suggest that adjuvants targeting dendritic cells are useful in peptide-based vaccines. Indeed, a new strategy (TriVax) based on the administration of co-stimulatory anti-CD40 monoclonal, TLR agonist Polyinosinic-polycytidylic acid [Poly(I:C)] and CD8+ T-cell epitope HPV 16 E7 (aa49-57) was able to induce tumor clearance in two HPV-induced murine cancer models [57]. Many of these protein/peptide-based vaccines moved to clinical trials where all of them indicated low toxicity and a good safety profile, but a strong discordance exists between immune and clinical responses, reinforcing the need of further improvement to the vaccination.

However, among the protein-based vaccine candidates, SGN-00101 vaccine, a fusion protein consisting of Hsp from Mycobacterium bovis and HPV 16 E7, has generated considerable interest. As a single-agent therapy, in both phase I and phase II clinical trials, fusion protein was able to induce regression of lesions in anal high-grade squamous intraepithelial lesions [58], recurrent respiratory papillomatosis [59], and CIN 2-3 [60-62]. In addition, phase II clinical trial with TA-CIN, a fusion protein-based vaccine expressing HPV 16 L2-E6-E7 conjugated proteins, in conjunction with topical application of TLR agonist Imiquimod, an imidazoquinoline amine, showed high levels of CD4+ and CD8+ T cells locally in patients with high-grade vulvar intra-epithelial neoplasia (VIN) [63]. One year after treatment 63% patients showed complete clinical regression of VIN lesions, associated with HPV clearance in 36% of the subjects. More recently Genticel’s vaccine candidate Procervix utilizing the adenylate cyclase (cyaa) technology, a protein vector that delivers the E7 antigens from HPV 16 and HPV 18 was proved safe in phase I trial. The company is now (March 2014) launching its phase II trial in women infected with high-risk HPV before the appearance of high- grade cervical lesions. This trial is the first ever recruiting HPV infected women with no cervical lesions and it would give information about a vaccine that may close the gap between preventive vaccines and later stage therapeutic options [64].

Since peptide-based vaccines are stable, easy to produce and have a high safety profile, many clinical trials have been reported. Research has focused on addressing the main limitations of peptide-based vaccines, namely their low immunogenicity, and the enhancement of the immunogenicity of peptide-based vaccines has been explored in clinical studies. The PADRE universal T-helper peptide was utilized to increase the activity of CTL epitopes encoding HPV 16 E7 that was presented by HLA-A*0201 (50% of the general population). These vaccines failed to mount a valid immune response in women with late stage cervical cancer [65,66]. More promising results were obtained in HLA-A2-positive patients with CIN/VIN 2/3 [67], where HPV E7 lipopeptide (aa 86-93)/PADRE was able to stimulate an immune response and led to complete regression of CIN lesions in 3 of 17 valuable patients. However, these vaccines presented limitations due to the HLA restriction. Therefore, this limitation was avoided by using long peptide which prompted their utilization in clinical trials. In cervical cancer patients who had undergone resection, the use of immunization with 13 overlapping long peptides spanning the entire sequence of HPV 16 E6 and E7 mixed with Montanide ISA 51 clearly revealed immunization-driven IFN-gamma production in enzyme-linked immunospot (ELISPOT) assay after completing the protocol [68]. When this same platform was tested in immunizing cervical cancer patients with active disease, both CD4+ and CD8+ T-cell IFN-gamma responses were detected toward both antigens [69]. Moreover, significant increases in proliferative capacity were also noted in responding T cells, reminiscent of the type of response noted in spontaneous regression [69]. This vaccine was well tolerated with few side effects: minor swelling at the injection site and flu-like symptoms. Phase II clinical trials of this vaccine in histologically confirmed HPV 16-positive high-grade VIN patients had a complete regression of their lesion after 3 or 4 vaccinations with HPV 16 E6/E7 overlapping peptide vaccine [70]. In the non-responders to the vaccine, an increased number of HPV 16-specific CD4 + CD25 + Foxp3+ Treg cells was ascertained [71].

The presence of these Foxp3+ T cells is linked to impaired immunity in malignancies. The efficacy of this vaccine was also demonstrated in a placebo-controlled randomized Phase II study showing an increased number of HPV 16-specific T cells in patients with HPV 16+ high squamous intraepithelial lesion (HSIL) [72].

### Plant-derived/produced vaccines

Plant molecular pharming represents a well-established biotechnology area that includes the production of protein biopharmaceuticals such as enzymes, hormones, antibodies, and vaccine antigens in plant systems. Plant-produced proteins represent a significant fraction of pharmaceuticals in advanced preclinical and clinical trial status. However, plant platforms present several drawbacks: time-consuming in generating stable transgenic lines, non homogeneous protein production in different tissues, impact of pests and diseases even in controlled conditions (greenhouses) and, more importantly, growth in non-sterile conditions. This last point may affect the good manufacturing practices (GMP) necessary for the production of pharmaceuticals. Transient expression or in vitro culture have emerged as alternative platforms to circumvent some of these drawbacks. Food and Drug Administration (FDA) has recently approved the first plant-made drug for human use, an enzyme produced in genetically engineered carrot cells for treating type 1 Gaucher’s disease. Plant production of candidate prophylactic and therapeutic HPV vaccines is proven, with evidence of efficacy in animals. There are data showing that an adjuvant-like effect was obtained in immunizations with crude tobacco plant extracts containing the E7 protein of HPV 16 [73,74]. The recombinant plant-derived vaccines as ‘in planta formulation’ without adjuvants were able to elicit also a protective Th1 cell response in mice. Similar adjuvating activity was seen in another tobacco plant-produced fusion protein of the HPV 16 E7; this antigen preparation was able to induce a specific CD8+ T stimulation that elicited a therapeutic effect on experimental tumors [46,47]. Finally, the possibility to produce E7 with high immunological activity in microalgae opens the way to producing antigens at affordable price, retaining the adjuvating activity of these plant-derived antigens [75]. An FDA-approved clinical trial for non-Hodgkin’s lymphoma with plant-produced single-chain variable fragment (scFv) was able to establish the safety and immunogenicity of plant made human vaccines [76,77], thus indicating the feasibility of this approach for human anticancer therapies. However, to our knowledge, no clinical trial with plant derived anti-HPV vaccine has been carried out yet.

### DNA/RNA based vaccines

DNA vaccines have been used in the clinical arena to elicit antigen-specific immune responses. Although nucleic acid vaccines do not appear to induce as vigorous immune responses as live viral vaccine vectors, they have several advantages, mainly naked DNA is relatively safe, stable, cost efficient, and able to sustain reasonable levels of antigen expression within cells. DNA-based plasmid vectors remain stable in a wide range of conditions over great lengths of time (longer periods than RNA vaccines), and they can be delivered with little risk to individuals who are immunosuppressed. In addition, since DNA vaccines do not elicit neutralizing antibodies in the vaccinated patient, they can be repeatedly administered with similar efficacy. Many strategies have been employed to produce an efficient delivery of targeted antigen-to-antigen presenting cells (APC) such as dendritic cells (DCs), an enhancement of antigen processing and presentation in DCs, and an augmentation of DC and T cell interaction [78]. Recently, it has been reported that the fusion of the E7 gene of HPV 16 with a plant virus coat protein produced strong antitumor activity in a mouse model activating both CD4+ and CD8+ T cells [46,79], as well as a fusion of E7 gene to a gene encoding a mutated form of the immunotoxin from saponaria officinalis, the saporine [80]. The latter should enhance the activity of the vaccine by the immunomodulant activity of this mutated saporine that has lost its toxic activity. The possibility to utilize enhanced delivery methods like electroporation, microencapsulation, and gene gun has further enhanced the targeting of DNA vaccine to DCs.

A dose-escalation trial of plasmid DNA encoding a transgene that produced E7 linked to Hsp70 showed limited efficacy at the highest dose, with low induction of responses in the IFN-gamma ELISPOT assay and a resolution rate of 33% [81].

A plasmid DNA encoding a 13-amino acid sequence of E7 encapsulated in biodegradable poly (D,L-lactide-co-glycolide) micro-particles was utilized to develop the ZYC101 vaccine expressing a HPV 16 E7 HLA-A2 restricted peptide. Two different phase I clinical trials examining the potential treatment of patients with anal dysplasia or with high-grade CIN, respectively, demonstrated a high number of immunological response i.e. circulating HPV-specific T cells and histological regression/improvement in 1/3 of the patients [82,83]. The improved version ZYC101a, that includes in addition to the HPV-encoding sequences of HPV 16 E7 the regions encoding segments of HPV 16 and HPV 18 E6 and E7 viral proteins, is one of the few therapeutic vaccines reaching the phase II/III in clinical trials involving subjects with high-grade CIN. In a prospectively defined population of women younger than 25 years CIN resolution was significantly higher in the ZYC101a groups compared to placebo [84]. Its activity in the treatment of patients with CIN 2/3 was also evaluated in a double-blinded, randomized, placebo-controlled clinical trial where half of 21 patients receiving the vaccine showed HPV 16/18-specific T cell responses but only 6 patients recovered from the high grade CIN [85].

VGX-3100, a DNA vaccine incorporating plasmids targeting HPV 16 and 18 E6 and E7 proteins was utilized in clinical trials employing electroporation technology. This technology was demonstrated particularly effective in animal models where the candidate vaccine is delivered via intramuscular injection followed by electroporation using various devices to deliver a small electrical charge. In a phase I clinical trial, 78% of the VGX-3100 vaccinated high-grade CIN subjects showed T cell and antibody responses [86]. On the bases of these findings, a double-blinded, randomized, placebo-controlled phase II clinical trial is ongoing on high grade CIN (NCT01304524). Further trials will be developed associating this vaccination with IL-12, used as an adjuvant, in order to significantly increase the CD4+ T cell response and with a vaccination against telomerase (hTERT), an antigen that is known to be related to a great number of human tumors and whose immunogenicity was demonstrated by the presence of existing naturally occurring T cell responses (Bagarazzi M. Personal Communication).

Other DNA vaccines have also been associated with other adjuvating treatments. In particular, the TLR7 agonist, Imiquimod promoting the activation of antigen-presenting cells and, in turn, leading to the production of cytokines IFN-alpha, IL-6, and TNF alpha [87] was shown to be active in mouse models [88]. In particular, the Imiquimod treatment affected the tumor microenvironment by reducing the number of myeloid-derived suppressor cells that have an immunosuppressive role and increasing natural killer (NK) and NKT cells that may play a role in tumor volume reduction. Thus this approach was utilized in an ongoing phase I clinical trial, which investigates a prime-boost strategy, combined with topical Imiquimod in treating patients with CIN3. The prime-boost strategy consisted of a DNA vaccine encoding an endoplasmic reticulum signal sequence (Sig), linked to an attenuated form of HPV 16 E7 fused to Hsp70 (pNGVL4a-Sig/E7(Detox)/Hsp70) boosted with a recombinant vaccinia virus encoding E6 and E7 of HPV 16 and 18 (TA-HPV) [NCT00788164].

Finally, the use of RNA replicons is a potentially interesting strategy for HPV vaccination. RNA replicons are naked RNA molecules derived from alpha-viruses, such as Sindbis virus [89,90], Semliki Forest virus [91-93], and Venezuelan equine encephalitis (VEE) [94] viurs. These RNA vaccines are self-replicating and self-limiting, and may be administered as either RNA or DNA, which is then transcribed into RNA replicons. RNA replicon-based vectors can replicate in a wide range of cell types and can be used to produce sustained levels of antigen expression in cells, making them more immunogenic than conventional DNA vaccines. However, RNA replicons are less stable than DNA. To combine the benefits of DNA and RNA replicon, DNA-launched RNA replicon, termed ‘suicidal’ DNA was utilized for HPV vaccine development in preclinical models [94,95].

This ‘suicidal DNA’ is transcribed into RNA within the transfected cell and provides a stable and efficient way to express tumor antigen but the cells may undergo apoptosis. Another replicon system is derived from the flavivirus Kunjin (KUN) [96]. The new generation of KUN replicon vectors, which allows for the synthesis of replicon RNA from plasmid DNA did not induce cellular apoptosis and was able to elicit specific T cell responses protecting mice from tumor challenge [97]. However, despite the general success of RNA replicons in preclinical models, RNA replicon-based vaccines have had limited clinical testing.

The newest mRNA-based vaccines have also been developed by Novartis as a non-viral delivery system for self-amplifying RNA that has been successfully tested in preclinical models. Another mRNA-based vaccine is the RNActive® vaccine platform from CureVac (Tübingen, Germany) that is based on a more stable modified mRNA sequence with increased immunogenicity by complexation with protamine. This mRNA vaccine exploits both the antigenic and the adjuvant properties of mRNAs to activate the adaptive and innate immune system. Two ongoing clinical trials on patients with prostate and non-small cell lung cancer show that RNActive® vaccines are safe and effective in inducing long lasting, humoral and cellular immune responses. No information is available on the possible utilization on HPV-associated cancers.

### Bacterial/viral vectors

Bacteria, such as Listeria monocytogenes (LM) [98,99], Lactococcus lactis [100], Lactobacillus casei [101], Salmonella and Bacillus Calmette-Guerin, and several viral vectors, including vaccinia virus (VV), adenovirus, adeno-associated virus, alpha-virus, and its derivative vectors, have been used to deliver genes or proteins of interest to elicit antigen-specific immunotherapy. Among the bacterial vectors, LM has emerged as a promising vector, because in animal models it is able to induce both CD8+ and CD4+ immune responses, to elicit regression of established tumors, and to overcome central tolerance by expanding low avidity CD8+ T cells specific for E7 [98]. ADXS11-001 a live, attenuated LM bacterial vector secreting HPV 16 E7 fused to listeriolysin O (LLO) LLO was utilized in clinical trials. Promising results of this vaccine in phase I trials for safety and immunological responses [102,103] were further assessed in phase II clinical trials. At least three trials are ongoing involving women with persistent or recurrent cervical carcinoma (NCT01266460), with CIN 2/3 with surgical indication (NCT01116245) [104], and patients (including male) with HPV-associated oropharyngeal cancer (NCT01598792).

Among viral vectors employed for the expression of HPV antigens, like adenoviruses [105,106], alpha-viruses [107-109] and VV [110-112], the last virus was historically one of the first viral vectors employed in clinical trials on therapeutic vaccines against HPV-associated cancer [113]. More recently Avipox viruses have been developed as novel vectors for the development of vaccines. Although their replication is restricted to avian they are permissive for entry and transgenic expression in most mammalian cells, and immunologically non cross-reactive with vaccinia, which avoids pre-existing immunity in smallpox experienced humans. Avipox viruses might therefore represent safer immunogens that have demonstrated their activity in inhibiting the growth of HPV16 E7 expressing tumor in C57 Bl6 mice with a HPV16 E7 DNA-prime/Fowlpox HPV16 E7-boost schedule [114].

To date many VV vaccines have been employed in clinical trials to deliver genes and antigens of interest efficiently. Phase I/II clinical trials in patients with vulvar or vaginal and early or late -stage cervical cancer were conducted with a vaccinia vector encoding HPV 16 and HPV 18 E6 and E7 antigens (TA-HPV) recombinant VV [113,115-117]. In particular, in a phase II clinical trial, 29 patients with stage I or II cervical cancer were vaccinated twice via scarification with TA-HPV; although clinical outcomes were not measured due to surgical intervention in all patients, induction of CTL responses were detected in a number of patients in the form of target cell lysis by isolated peripheral bone marrow cells (PBMCs) [117].

In another study, a recombinant VV expressing E6 and E7 antigen together with IL-2 (TG4001/R3484) was administered to CIN 2/3 patients, with very promising clinical results. Ten patients (48%) were evaluated as clinical responders at month 6. At month 12, 7 out of 8 patients without conization reported neither suspicion of CIN 2/3 relapse nor HPV 16 infection [118]. Interim results phase IIb trial on patients with HPV-related CIN 2/3 lesions demonstrated the activity of vaccine in monotherapy, but the trial did not reach its primary endpoint of six-month resolution of CIN 2/3 and will not move on to a phase III trial [119]. A recombinant modified vaccinia Ankara vector was also utilized to express papillomavirus protein other than E6 or E7, namely the bovine papillomavirus E2 (MVA-E2). E2 is a transcriptional repressor of E6 and E7 oncogenes, it was assumed that in low grade lesions, where the virus is not integrated, the MVA-E2 expressed protein could bind to the repressor region of the viral genome. However, there is no evidence for E2 expression direct contribution to the therapeutic effect seen in both patients with CIN [120,121] and genital warts [122]. The vaccine could be active such as a non-specific “pro-inflammatory stimulation” in the cervico-vaginal tract that induces an immune response.

Finally, in alternative to viral vectors, synthetic viral vector like virus like particle (VLP) can be utilized because they are easy to manufacture and have the capacity for compacting DNA, and targeting specific cell receptors. Thus, the same technology used for producing anti-HPV prophylactic vaccines was employed for producing chimeric VLPs. An L1–E7 fusion protein has been shown to self-assemble into chimeric VLPs (CVLP) that can induce E7-specific cellular immunity in mice [123]. A randomized, double blind, placebo-controlled clinical trial has been conducted in CIN 2/3 patients with CVLP. Antibodies with high titers against HPV 16 L1 and low titers against HPV 16 E7 as well as cellular immune responses against both proteins were induced. Although not statistically significant a trend for histological improvement to CIN I or normal histology was seen in 39% of the patients [124].

### Dendritic cells (DCs) based vaccines

The immune response to initial stages of infection causes inflammatory responses that trigger innate effector cells, such as NK and NKT cells. This inflammatory response, driving the innate immunity, is initiated through pathogen-associated molecular pattern (PAMP) sensors including TLRs 1–9. These receptors in response to specific bacterial or viral components activate APCs via the transcription factor nuclear factor KB (NF-KB). In addition, infection may alter the local metabolic and cellular microenvironment activating danger-associated molecular pattern (DAMP) sensors, particularly nucleotide-binding oligomerization domain (NOD)-like receptors (NLRs), as components of multiprotein complexes termed the inflammasomes, inducing maturation and releasing members of the IL-1 family. In particular, the produced IL-1b and IL-18 mediate repair responses such as angiogenesis and, via upregulation of cytokines and chemokines, induce the recruitment of inflammatory cells to the site of infection. The observed slow clearance of HPV infection and weak immune responses to viral proteins is a likely consequence of the nonlytic nature of HPV infection and a consequent delay in induction of PAMP- and DAMP-induced inflammatory responses through TLRs and the inflammasomes. Thus, in the absence of inflammation a number of events take place (i.e. IL-10 production by Th cells and mast cells; IFN-gamma production by CD-1d-activated NKT cells, increased TGF-beta) inducing negative regulatory signals that in turn can change the state of the APC by altering costimulatory molecule expression, thus inhibiting induction of cytotoxic effector T cells. Therefore a therapy aimed to reactivate these APCs could be a valid tool for clinical intervention.

Among specialized APCs the most potent are DCs, because they express high levels of MHC and co-stimulatory molecules. Therefore, DCs were the research focus of many investigators and a variety of methods for generating DCs, loading them with tumor antigens, and administering them to patients. The success of Provenge, a DC vaccine incorporating prostatic acid phosphatase, in patients with advanced prostate cancer has generated great interest for DC-based vaccines [125,126]. Although expensive, DC-based vaccines have been tested in patients with HPV-associated cervical cancer by successfully transducing genes coding for E6 and E7 into DCs. In a clinical study, autologous DCs were pulsed with HPV 16 or HPV 18 E7 recombinant proteins and E7-specific CD8+ T cell responses were observed in 4 out of 11 late stage cervical cancer patients [127]. In another clinical study, stage IB or IIA cervical cancer patients were vaccinated with autologous DC pulsed with recombinant HPV 16/18 E7 antigens and keyhole limpet hemocyanin 1 (KLH), an immunological carrier protein. This vaccine generated E7-specific T cell responses in 8 out of 10 patients and antibody responses in all patients [128]. Another ongoing clinical trial is being conducted in Taiwan National University in recurrent cervical cancer (NCT00155766), using DCs pulsed with HPV 16 E7 antigen. However, DC-based vaccines could be used to treat advanced cervical cancers but are unlikely to be used to treat CIN lesions because the procedures involved in this kind of treatment are expensive and labor-intensive.

## Combinational immunotherapy

Given the importance of local microenvironment in the persistence of HPV lesions, strategies aiming to alter local immunity have shown some positive results, therefore therapeutic HPV vaccine strategies have shifted toward combinatorial approaches with radiotherapy and chemotherapy. Low-dose radiation in combination with HPV vaccination was effective in the treatment of tumors in preclinical models [129]. Radiation therapy seems to be a useful method in stabilizing tumor cell growth when applied with immunotherapy by inducing apoptosis in tumor cells.

A chemotherapeutic agent in combination with DNA-based vaccines was proven an effective HPV therapy in preclinical models [130-136]. Low cyclophosphamide doses, altering local immunity, exerted some positive effects in persistent low-risk HPV lesions [135]. A randomized trial comparing chemotherapy versus a combination with the HPV 16 Synthetic Long Peptide (SLP) in advanced cervical cancer is planned. A pilot study is ongoing with the aim to investigate the optimal time (“window”) for the vaccination with the HPV 16 SLP vaccine after standard treatment of 6 cycles Carboplatin-Paclitaxel every three weeks (EudraCT2010-018841-76).

A randomized study was carried out in 110 recurrent/refractory cervical cancer patients with cisplatin and different doses of HPV bacterial vector-based vaccine ADXS11-001, and preliminary results showed efficacy and manageable toxicity [137].

In addition, other compounds affecting the immunological environment like COX-2 inhibitors, through the prevention of the production of prostaglandin E2 or antibodies to IL-6 [138] or IL-10 [139] or the TLR agonist Imiquimod could be effective.

In particular, Imiquimod is already in clinical use against warts stimulating local innate immunity and potentiating adaptive immune response by activating tissue antigen presenting cells. A number of studies with topical Imiquimod have been reported with favorable results mainly in vulvar intraepithelial neoplasia (VIN) lesions [63,140] and combination therapy with HPV DNA vaccines described in the previous paragraphs of this review.

Cytokines-based therapies in combination with HPV therapeutic vaccine showed promising results in preclinical models. Treatment with IL-12 gene, administered as gene therapy, viral gene therapy, by adenovirus, and in combination with E6-E7 oncogenes, determined tumor growth suppression [141,142].

Recently, programmed death-1 receptor (PD-1) is emerging as a pivotal target for combination therapy by affecting immune suppression.

Indeed, PD-1 is expressed on T cells following T-cell receptor (TCR) activation and binding of this receptor to its cognate ligands, programmed death ligand (PDL)-1 and PDL-2, down-regulates TCR signals, promoting T-cell anergy and apoptosis, thus leading to immune suppression.

An anti-PD-1 antibody (CT-011) with Treg-cell depletion by low-dose cyclophosphamide (CPM), combined with HPV 16 E7 peptide vaccine, produced synergistic antigen-specific immune responses inducing complete regression of established tumors in a significant percentage of treated animals, with prolonging survival [143].

Expanded phase I clinical studies with anti-PD-1 (BMS-936558) and anti-PDL -1 (BMS-936559) showed objective clinical responses in renal cell carcinoma, melanoma, and non–small cell lung cancer, and a relationship between tumor cell surface PD-L1 expression and objective responses to anti-PD1 therapy [144,145]. In addition, a recent study showed that PD-1: PDL-1 pathway may create an “immune-privileged” site for initial viral infection in the tonsils and subsequent adaptive immune resistance once tumors are established suggesting a rationale for therapeutic blockade of this pathway in patients with HPV + oropharyngeal squamous cell carcinoma [146]. Other strategies trying to inhibit the suppressive tumor microenvironment utilize monoclonal antibodies such as Ipilimumab. This antibody is a fully human monoclonal antibody against the cytotoxic T-lymphocyte antigen-4 (CTLA-4), an immune-inhibitory molecule expressed in activated T cells and in suppressor T regulatory cells. The interaction between the monoclonal antibody and CTLA-4 blocks inhibitory signals and enhances T cell activation, leading to increased antitumor responses [147]. Ipilimumab has been approved for melanoma, but ongoing trials are testing the drug in other tumors, among them locally advanced cervical cancer, in a sequential regimen following chemoradiation (NCT01711515).

## Conclusions

The development of cervical cancer depends not only on efficient negative regulation of cell-cycle control supporting the accumulation of genetic damage, but also on a sophisticated viral mechanism of immune evasion [20-22]. Despite this complex interplay, most HPV infections are cleared within one year, and cell-mediated immunity plays an important role in this process. Thus, a better understanding of immunological path involved in cervical cancer carcinogenesis is an important goal in cervical cancer knowledge, and the development of new treatment modalities involving immune system may represent an innovative strategy in this complex disease. Although antigen-specific immunotherapy induced by therapeutic HPV vaccines was proved extremely efficacious in preclinical models, its progression through clinical trials suffered of the early poor responses. Such initial clinical trials were conducted in advanced cancer patients to evaluate the safety of the respective vaccine. Because of the immunosuppressive milieu generated by established tumors, it appears that (likewise to cancer immunotherapy in general) in cases of advanced disease, an immunological antitumor effect will, if at all, only be successful along with standard therapy. Later attempts took advantage of the unique situation in HPV-related carcinogenesis, that is, the existence of well-defined precursors to cervical, anal or vulvar cancer.

Several clinical studies on these precursors lesions have so far been completed with promising effects, even if these trials were not powered to detect small effects in the vaccine versus the placebo groups. Indeed, there was no follow-up study with the same strategy, perhaps as a result that these trials were investigator-initiated or sponsored by small biotech companies.

Large pharmaceutical corporations that could have ensured continuation of the individual program and guarantee the prosecution in more powered phase II/III trials were completely lacking. Even the partnership announced in 2007 between a large pharmaceutical firm (Roche) and a biotech company (Transgene) on a promising vaccine TG4001 was recently discontinued.

In conclusion immune escape and immune suppression are active in cancer and to some extent also in premalignant lesions and, therefore one might consider targeting persistent infections before they become clinically apparent. This ‘paradigm shift’ in vaccination of cancer patients is a quite logical step in HPV-related cervical cancer. Treating this early condition with post-exposure prophylaxis might be more successful than therapy at more advanced stages. Several well-defined cohorts of women with persistent infections do exist, and the design and execution of clinical trials aiming to clear of HPV DNA should be straightforward. However, it is important to take into consideration that patients with HSIL are generally asymptomatic and can be treated by minor surgical procedures. A clinical trial on long overlapping peptide vaccine in CIN 2/3 patients was stopped prematurely because motivational problems of the patients and the local/systemic side effects of the vaccine. Thus, motivational problems must be taken into account when considering studies in patients with premalignant lesions for whom an effective treatment is available [72]. Future developments should consider the disparities of side effects between standard of care and new therapies to maximize the potential benefits of therapeutic vaccination. In addition, therapeutic efficacy would receive beneficial effects by addressing the immunosuppressive tumor microenvironment. Combinational strategies particularly for cancer patients are a promising tool to control tumor metastasis and eliminate cancer cells altogether.

The development of prophylactic and therapeutic HPV vaccines must be continued in order to come close to the eradication of HPV-associated malignancies even in other localizations [148,149], getting rid of the perception (after the effectiveness of commercial preventive vaccines) that the problem has been solved.

## Competing interests

The authors declare that they have no competing interests.

## Authors’ contributions

The outline was conceived by AV. All authors contributed to initial drafts, edited version, and the final version. All authors read and approved the final manuscript.
